# Towards a paradigm shift in environmental health decision-making: a case study of oxybenzone

**DOI:** 10.1186/s12940-021-00806-y

**Published:** 2022-01-08

**Authors:** Klara Matouskova, Laura N. Vandenberg

**Affiliations:** Department of Environmental Health Sciences, School of Public Health and Health Sciences, University of Massachusetts – Amherst, 171C Goessmann, 686 N. Pleasant Street, Amherst, MA 01003 USA

**Keywords:** Sunscreen, Endocrine disruptor, Skin cancer, Benzophenone 3, Intergenerational, Externality, Melanoma, Conflicting interests

## Abstract

**Background:**

Technological advancements make lives safer and more convenient. Unfortunately, many of these advances come with costs to susceptible individuals and public health, the environment, and other species and ecosystems. Synthetic chemicals in consumer products represent a quintessential example of the complexity of both the benefits and burdens of modern living. How we navigate this complexity is a matter of a society’s values and corresponding principles.

**Objectives:**

We aimed to develop a series of ethical principles to guide decision-making within the landscape of environmental health, and then apply these principles to a specific environmental chemical, oxybenzone. Oxybenzone is a widely used ultraviolet (UV) filter added to personal care products and other consumer goods to prevent UV damage, but potentially poses harm to humans, wildlife, and ecosystems. It provides an excellent example of a chemical that is widely used for the alleged purpose of protecting human health and product safety, but with *costs* to human health and the environment that are often ignored by stakeholders.

**Discussion:**

We propose six ethical principles to guide environmental health decision-making: principles of sustainability, beneficence, non-maleficence, justice, community, and precautionary substitution. We apply these principles to the case of oxybenzone to demonstrate the complex but imperative decision-making required if we are to address the limits of the biosphere’s regenerative rates. We conclude that both ethical and practical considerations should be included in decisions about the commercial, pervasive application of synthetic compounds and that the current flawed practice of cost-benefit analysis be recognized for what it is: a technocratic approach to support corporate interests.

## Introduction

**Fig. 1:**
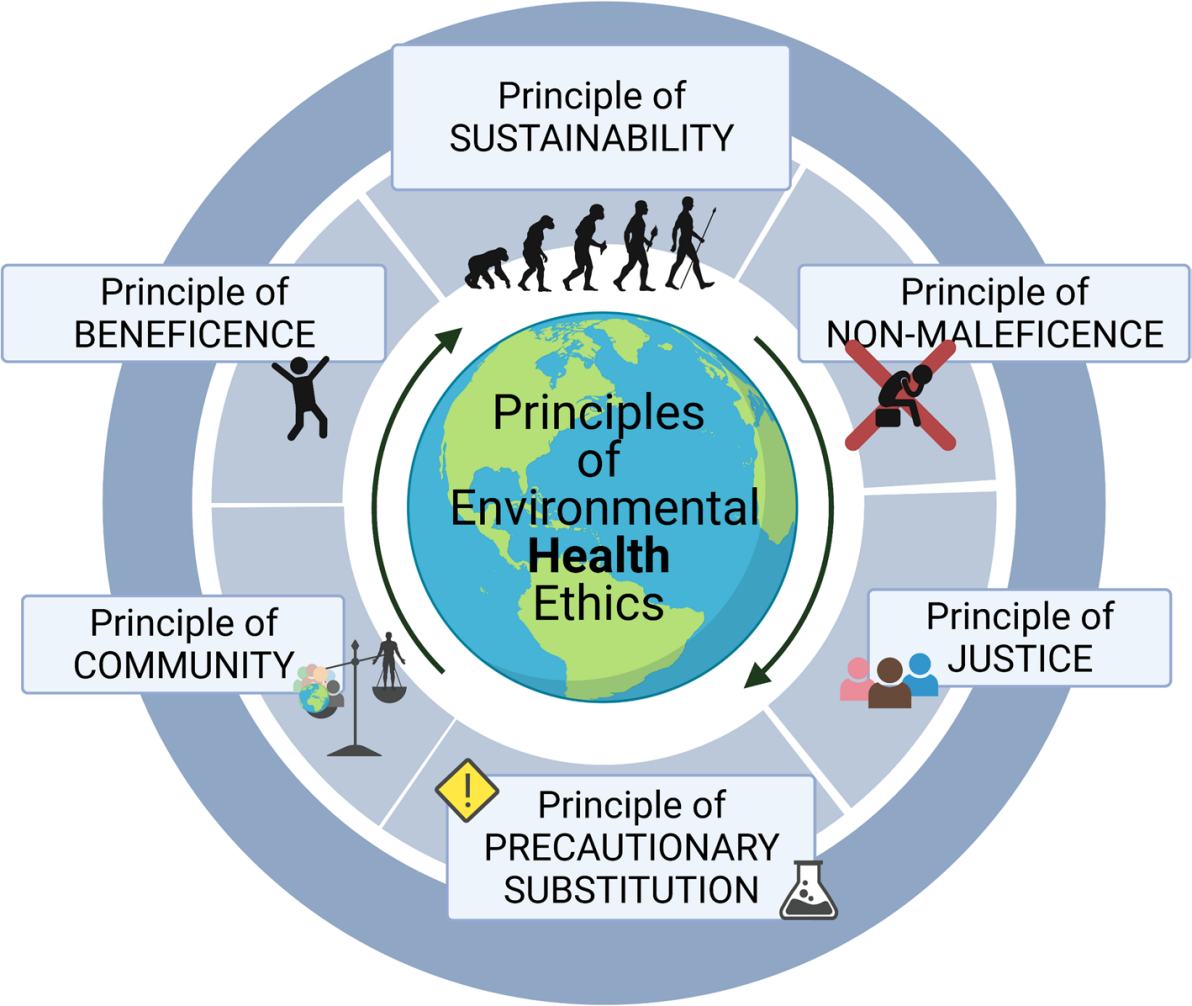
Six proposed principles of environmental health ethics. Ethical principles of sustainability, beneficence, non-maleficence, justice, community, and precautionary substitution provide a framework that can be used to evaluate environmental chemicals. This framework allows for decision-making about synthetic compounds beyond the traditional cost-benefit analysis

Thousands of (mostly inadequately tested) synthetic chemicals are currently on the market [1]. Some of these compounds are intended to increase human safety, while others enable medical interventions, and still others provide human conveniences. For better or worse, technological and chemical advances of the last century have improved and eased individual and public well-being. Yet, these advances do not come without a cost to the environment, other species and, somewhat paradoxically and with unintended consequences, to human health. For example, phthalates added to flexible tubing (an important feature of medical equipment) also interfere with male reproduction [2]. The conflict between the benefits of safety or convenience versus the unexpected cost to human health is, however, only one of the many controversial facets of decision-making pertaining to environmental chemicals. Environmental health decision-making also extends to the identification of economically and environmentally sustainable solutions, exercises precaution and prevents “plausible threats”, avoids quick fixes involving regrettable substitutions, addresses disproportionate impacts of environmental burdens on communities, and considers individual rights and obligations including the rights of individuals to know (or not know) about their environmental exposures [3]. Here, we propose six principles of environmental health that can assist in environmental health decision-making relative to environmental chemicals (Fig. 1).

## Introduction to Oxybenzone

**Fig. 2:**
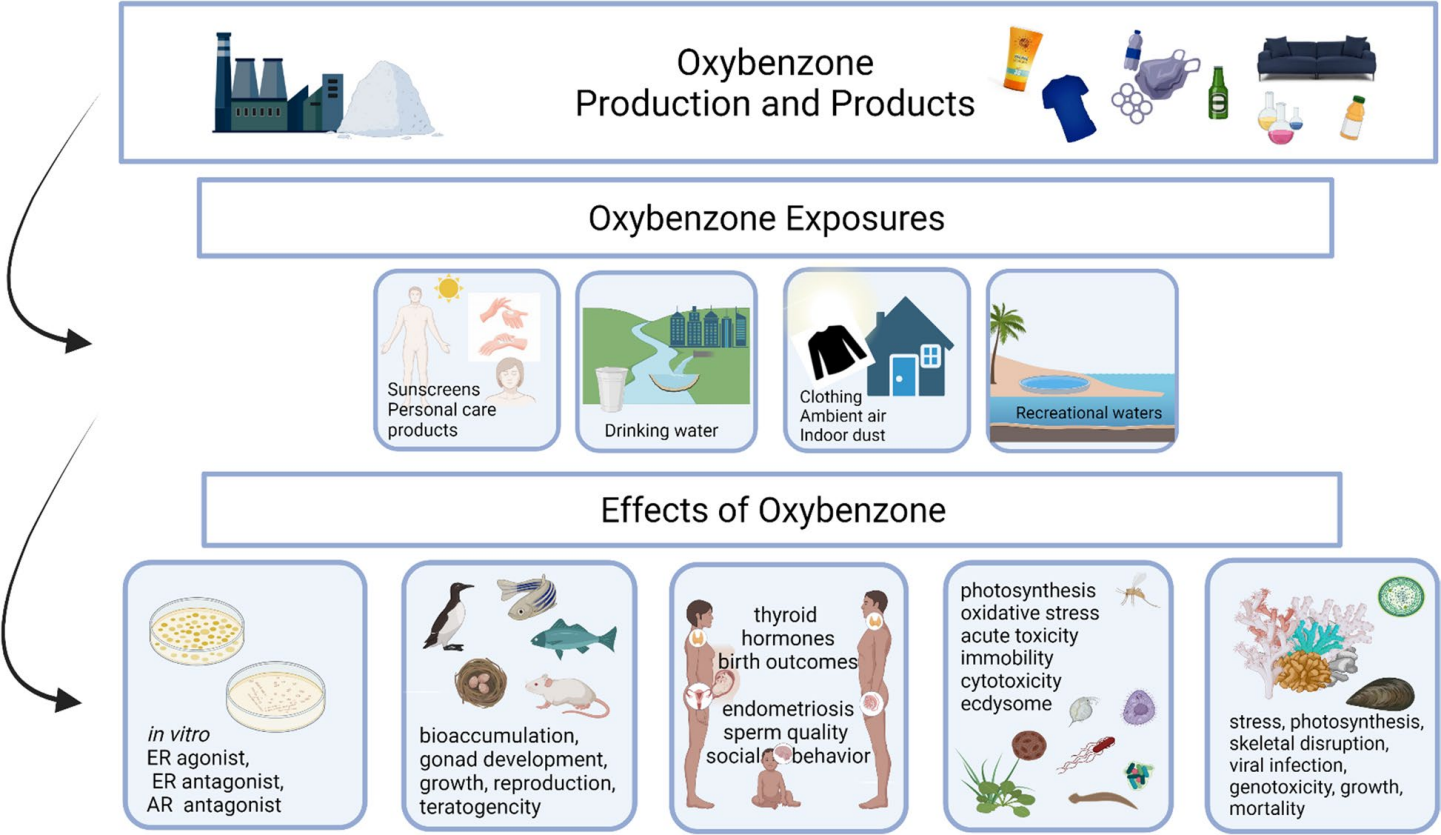
An overview of the oxybenzone case study. Owing to the production and manufacture of products containing oxybenzone, it is released into the environment, contributing to ubiquitous human exposures. Exposures are associated with a range of hazards to wildlife, laboratory animals and human health

To illustrate the framework of the proposed principles, we evaluated oxybenzone, a synthetic UV filter used in sunscreens and other personal care products; it is also added to many consumer products including cardboard inks, plastic packaging, fabrics, and furniture finishes to protect these commodities from UV-induced fading or damage [4] (Fig. 2). As a result of its popularity, oxybenzone is now among the most widespread environmental pollutants routinely detected in fish and avian tissues, plants and microorganisms [5–7]. Collectively, in humans, all sources of exposure contribute to detectable urinary concentrations in over 98% of the U.S. population [8]; it has also been measured in blood, amniotic fluid, cord blood, semen and breast milk [9, 10].

Recent randomized control trials revealed that oxybenzone reaches and exceeds the US FDA’s threshold of concern (0.5 ng/ml in blood) 2 hours after sunscreen application; these concentrations remained above the threshold for 23 h in all participants, and for 3 weeks in 96% of participants [11, 12]. Based on these new findings, the U.S. FDA recently requested additional safety studies on oxybenzone and other sunscreen ingredients [13].

The effects of oxybenzone in vivo are broad: it is toxic to cyanobacteria [14, 15], green algae [14, 16] and coral [17]. In rodents, oxybenzone alters the development of the mammary gland, alters the weight of the liver, kidney, and reproductive organs, decreases the number of spermatocytes in males [18–21] and induces DNA damage in the mammary epithelium [22]. In fish, oxybenzone interferes with reproduction [23, 24].

There is also evidence that oxybenzone is an endocrine disrupting chemical. In vitro screening tests have revealed that it is an estrogen receptor (ER) agonist and antagonist [25–27] and that some oxybenzone metabolites have greater estrogenic activity than the parent compound [28, 29]. Oxybenzone is also an androgen receptor (AR) antagonist [25, 26]. Consistent with AR antagonist activity, oxybenzone induces shortened anogenital distance in rats [20] and mice [19] following perinatal exposure (Fig. 2).

## Principles

*“It is essential my Son in order that you may go through this Life with comfort to yourself and usefulness to your fellow creatures that you should form and adopt certain principles for the Government of your own conduct and temper—unless you have such rules and principles there will be numberless occasions on which you will have no guide for your Government but your Passions…”*

J. Q. Adams [30]

Although principles originate from virtues conceived by the Ancient Greeks around fifth Century BCE if not earlier, the modern method of principlism in human health emerged during the late twentieth Century - initially as a response to atrocities of the first half of the century (e.g., human experimentation by the Nazi regime, the Tuskegee syphilis study, and others), later as reflecting emerging environmentalist movements [31]. In the field of ethics, principlism is considered a practical framework for “people making real-world decision[s]” [32]. Our everyday personal, societal, and professional conduct is guided by commonly accepted ethical principles: regardless of our religiosity, we shalt not murder or steal; democratic societies are ruled by consensus of “the people” rather than the will of an individual; and guilds and professional organizations declare their ethics guidelines and standards of practice.

Modern clinical medicine and related scientific disciplines have codified four guiding principles: non-maleficence (often translated as “do no harm”), beneficence, justice, and respect for autonomy [33]. However, the values and principles for public health or environmental health cannot be directly transferred from the field of medicine owing to differences in the type of interventions in public health vs. clinical practice (e.g., indirect and preventive vs. direct and curative), type of professionals involved (e.g., a diverse group vs. specialized), and the ultimate focus of interventions (e.g., to protect populations including unspecified individuals vs. a single well-known patient) [34]. Unlike medical ethics, public health ethics frameworks have not yet fixed upon a set of universally accepted principles; however, several foundational values, such as transparency, reduction of inequities, and solidarity run through many proposed frameworks. On the global end, a comprehensive document of sixteen principles, the Earth Charter, lays an ethical foundation for acting based on respect and justice, nonviolence and democracy, and ecological integrity [35–37].

Environmental *health* ethics (not to be confused with environmental ethics, which does not focus principally on the health of people) represents an approach where human health stands paramount in the context of and connection with the environment, non-human species, and possibly, future human generations. This may seem to present a tension between eco-centric and anthropo-centric approaches, but such dissention is anticipated and welcomed because applied ethics arises from epistemic uncertainty and works to address tensions. In environmental health ethics, an immediate focus on human health is extended towards non-human species, ecosystems and the biosphere, as well as the responsibilities of the human species that rises from its rights. Scholars of environmental health ethics have only begun discussing the guiding principles of the field [38]. In this context, the thousands of newly synthetized chemicals and the ubiquitous environmental pollution that is inevitably reaching human tissues have provided challenges to ethical decision-making and ethical actions.

### Principle of sustainability

*This we know: the earth does not belong to man; man belongs to the earth. All things are connected like the blood that unites all Man did not weave the web of life; he is merely a strand in it. What he does to the web, he does to himself.*

Sealth, Chief Seattle

Stewardship is broadly defined as a call to “take good care of natural resources” and implies additional subsidiary principles such as “protect species and biodiversity” and “avoid destruction of habitats and ecosystems” (p.73 [31]). That humans are not external to ecosystems and their biodiversity has been recognized by the global community [39, 40]. Nonetheless, responsible stewardship is still on occasion seen as binary– one may protect *either* nature *or* civilization - but like many issues presented as an either/or choice, this dichotomy is false. In fact, economists have repeatedly demonstrated that “the viability of business itself depends on the resources of healthy ecosystems” [41].

Acting as responsible stewards leads to a “practice of sustainable uses of biological resources” [31]. The *Principle of Sustainability* thus implies a balance of using - but not overusing; harvesting - but not overharvesting. Prior to the Anthropocene epoch, industrial chemicals would not present a threat because their volumes did not exceed the rate of individual and environmental biotransformation [42]. In the case of oxybenzone, this is no longer true. First synthesized in the mid-twentieth century, oxybenzone quickly gained dominance among sunscreen products and equally rapidly began polluting coastal waters when washed off the skin of beachgoers. The levels of oxybenzone measured in coastal waters harboring coral reefs are no longer sufficiently diluted to avoid harm to these species [17, 43]. The demand for sunburn-free midday beachgoing, combined with climatic and oceanographic conditions produce the present situation in which oxybenzone threatens the survival of aqueous species and ecosystems [44]. Although the chemical is readily metabolized in human tissues and 93% of the compound is transformed in the marine environment within 120 days of its introduction, oxybenzone is routinely detected in nearly all human samples, in water and soil, consistent with continuous pollution sources [45].

Although people have changed landscapes for the sake of utility, beauty, or leisure for most of human history, for millennia these changes played by nature’s rules simply because the members of our species were “few, humble, and weak” [46]. In recent history, however, technological and chemical discoveries have led to advances in which the rules of nature could be dismissed or ignored. Humans devised methods to defeat infectious diseases, to bypass infertility, and to stretch the limits of materials on water-repellency, flammability, and flexibility. With these changes, our civilization seemed invincible. Yet, in spite of these advances we have reached several “walls” where the limits of demography, physics and biology matter again. No longer can the biosphere transform all synthetic substances into harmless metabolites – there are tens of thousands of newly synthesized chemical compounds in the environment, many of them produced in large volumes, some of them highly stable and persistent in the environment [1]. After times when the elements have had extreme impacts on civilizations, and times when civilization has had extreme impacts on the environment, it becomes necessary to execute responsible and *sustainable* stewardship.

**Fig. 3:**
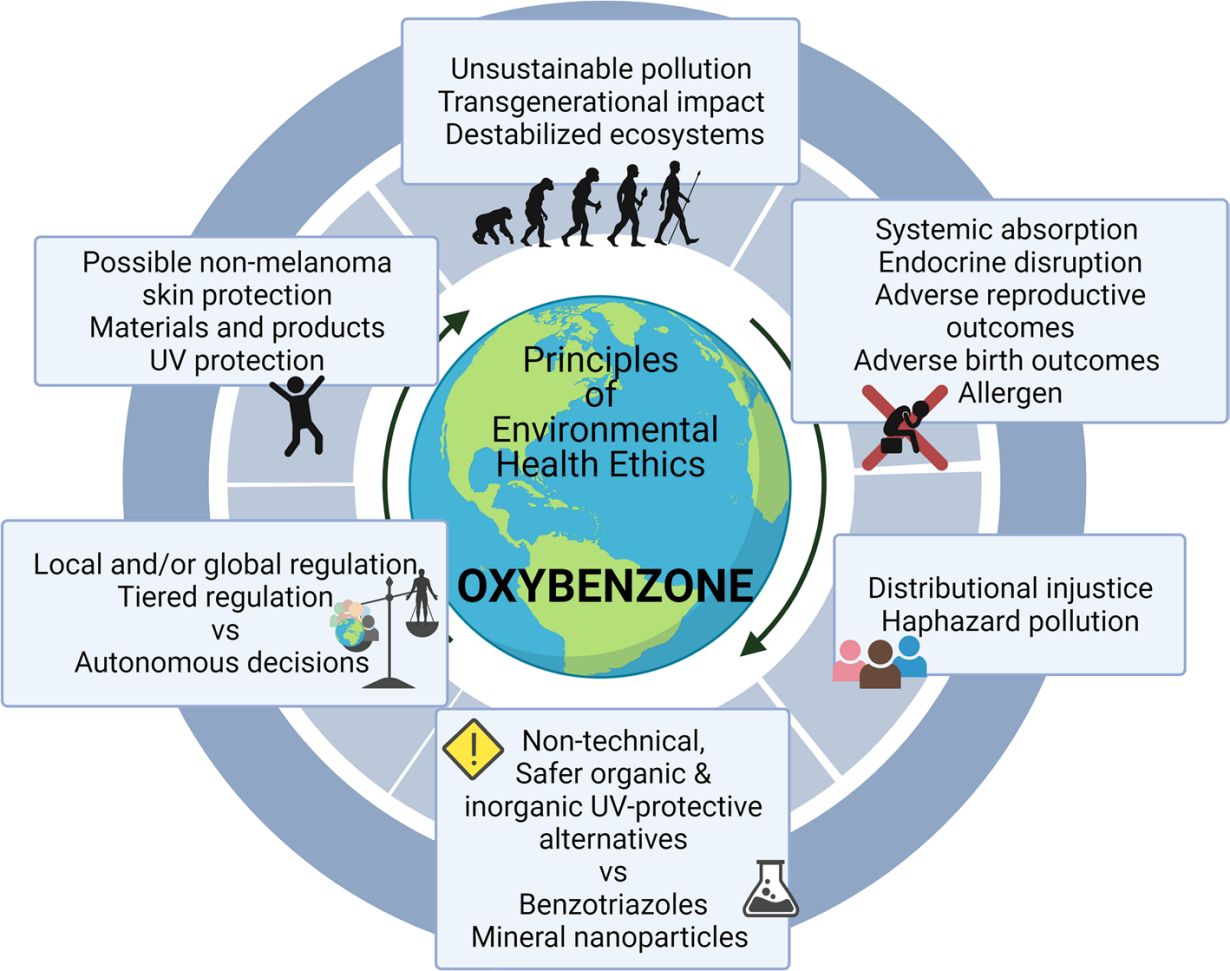
Evaluating oxybenzone based on the proposed six principles. Oxybenzone as a UV- absorbing filter in sunscreens, products, and packaging induces short- and long-term adverse effects on both humans and environment. Further, heavy coastal pollution stands as unequal and unjust. Systemic regulation on regional and global levels, tiered according to essentiality of use will affect autonomous decisions. On the other hand, alternatives to oxybenzone exist as: 1. non-technical solutions (preferred, when possible), 2. safer chemical UV filters (where necessary), and 3. inorganic UV-filters (to be used with caution)

Implied in the *Principle of Sustainability* is the matter of future generations. Given that coral reefs that form over thousands of years have shrunk by 20 to 80% over the past two generations, we are already past the turning point of our grandchildren experiencing the vast reefs as we did [47]. Some have argued that obligations to hypothetical future individuals is a paradox: how can “actions that make things worse for *no one* [in particular] be wrong?” [48]. The “non-identity case” solution most relevant to environmental health suggests replacing the hypothetical person of the future with an entire community potentially harmed by today’s action because “we have a moral duty to promote the overall well-being of future generations, even if we do not have moral duties to any particular future person” (p.74 [31]). *The Principle of Sustainability* weighs the impact of today’s production and use of anthropogenic chemicals along with the wellbeing of current and future humans, non-human species and the biosphere. Oxybenzone fails this test (Fig. 3).

### Principle of beneficence

*On one level, modern sunscreen isn’t so far from smearing yourself with clays, minerals, or a mixture of sand and oil like the ancient Egyptians or Greeks did. But on another level, modern sunscreens are some mind-bending magico-chemical spellwork. Our species should be patting ourselves on the back right now. But does our little magic trick actually work?*

George Zaidan, [49]

In the clinical setting, the *Principle of Beneficence* can be easily translated as actions that benefit the patient. Experimentally, chemical sunscreens have been clearly demonstrated to provide protection against UV-induced damage, and on the level of populations they were pledged to decrease skin cancer incidence [50, 51]. Therefore, the *Principle of Beneficence* raises a question: do chemical sunscreens actually offer a means of skin cancer prevention to at-risk populations?

The incidence of malignant keratinocyte carcinomas, i.e., squamous and basal skin cancers – previously referred to as non-melanoma skin cancers [52] – has increased by 14% between 2006 and 2012 with > 3 million U.S. residents affected in 2012 [53]. Melanoma cases are reported to cancer registries thus their statistics and trends are more precise: in 1975, the National Cancer Institute (NCI) recorded 7.9 cases of melanoma per 100,000 US residents; in 1995 the rate doubled to 16.5, and the latest data from 2018 report 22.4 melanoma cases per 100,000 residents [54].

Although their convenience is rarely disputed, the efficacy of sunscreens remains ambiguous. Whether sunscreens modify the risk of sunburn, melanoma and keratinocyte cancers have been studied predominantly using human cohorts, and the body of evidence provides a heterogenous and contradictory picture. In 2001, the International Agency for Research on Cancer published a review of case-control studies and concluded that the evidence for sunscreen providing protection from melanoma and basal cell carcinoma was “inadequate” while “limited” evidence for preventive effects was found for cases of squamous cell carcinoma [55]. A meta-analysis of 11 human cohorts found no effect of sunscreen use on sunburn prevention [56]. On the other hand, a French collaborative report concluded that “topical use of sunscreens reduces the risk of sunburn in humans” yet “no conclusion can be drawn about the cancer-preventive activity of topical use of sunscreens” [57]. A more recent meta-analysis included epidemiological studies beyond case-control designs, and carefully assessed the quality of evidence of sunscreen-melanoma associations; yet even this review finds the evidence inconclusive [58]. What is more, sunscreens seem to have no effect on melanoma risk in dark-skinned populations [59].

Only one study to date shows a protective effect of sunscreens (containing avobenzone and octinoxate) in an Australian randomized controlled trial [60]. However, individuals assigned to the “control” group were not restricted in their use of sunscreen, and the study was burdened with a number of biases, including selection and funding bias, and detected only half of the melanoma cases that were expected given incidence of the disease in the Australian population [61].

In spite of the paucity of efficacy evidence, humans mass-produce and promote sunscreens, and encourage an everyday, one-size-fits-all use. Since the early 1970s, guidelines of the American Association for Dermatology caution users to apply sunscreen on the “entire body before [a person] dress[es] for the day” and repeat applications throughout the day [62]; the goal for children and infants older than 6 months is “to protect all parts of the skin exposed to the sun by using a variety of techniques, including sunscreen” [63]. For many, everyday use of broad-spectrum sunscreens has become a matter of fact [64], but humans’ behavior sometimes works contradictory to the best public-health intentions. For example, there is evidence that application of sunscreen may increase sun exposure and a false sense of sun safety [65] by “extend[ing] the duration of intentional sun exposure, such as sunbathing… increas[ing] the risk for cutaneous melanoma” [57]. Lastly, sunscreen’s efficacy is subverted if users apply “however much feels right” [49], i.e. less than the recommended amount of 1 oz per application, or if a person fails to re-apply the product every 2 hours [66].

The *Principle of Beneficence* calls for convincing evidence and significant benefits to public health. The above arguments undermine the alleged beneficence of sunscreens, and oxybenzone specifically, in the prevention of skin cancer (Fig. 3).

### Principle of non-maleficence

*“Animal studies have raised concerns about endocrine disruption and reproductive issues. But animals are not people, [Dr. Henry] Lim [a dermatologist] says. And despite decades of sunscreen use, there has been no population-wide signal that rates of infertility, birth defects or other health problems are higher in people who use more sunscreen or in places where people apply more of it”* [67]*.*

“*Primum non nocere” (first, do no harm)* obligates health care professionals to abstain from inflicting harm to a patient if s/he cannot benefit from the care provided. The *Principle of Non-maleficence* builds on the argument of beneficence, and asks “is it safe for me”?

Sunscreens are regulated by the FDA and tested for sun protection efficacy. Yet, testing the 17 over-the-counter UV filters for safety (e.g., hazard identification) was previously not seen as necessary; the sunscreen ingredients, including oxybenzone, were considered by FDA to be “generally recognized as safe and effective” because it was assumed that their concentrations would not exceed a threshold of 0.5 ng/mL in systemic circulation after dermal application [13]. Now that this assumption has been shown to be wrong [11, 12], the FDA has called for more data and possibly stricter regulation [13].

Beyond concerns about exposure, research published in peer-reviewed journals paints a more complex story of possible health effects (e.g., hazards). An increasing number of epidemiological studies have evaluated the effects of oxybenzone on human health outcomes. Although many of these studies are limited by their design (e.g., cross-sectional studies), there is evidence that oxybenzone and its metabolites can affect human health, including effects on reproduction (time to pregnancy, risk of endometriosis, sperm quality, measures of infertility) [68–71]; birth outcomes [72, 73]; neurobehavioral outcomes in the offspring [74]; and health of other organs, e.g., the thyroid gland [75, 76]. Furthermore, oxybenzone was identified as an allergen in 1–3% of the population [77] and while allergic reactions to sunscreens affect only a small proportion of the population, oxybenzone is a common photoallergic agent [78]. In addition to the weight of epidemiological cues, as described in more detail above, mammalian toxicological studies similarly point to oxybenzone’s effects on the structure and function of tissues including endocrine organs [79].

Regardless, some dermatologists continue to promote chemical sunscreens as the ultimate means of sun safety and call for epidemiological studies to detect a “population-wide signal” for an effect of sunscreen use on human health before even entertaining the possibility that oxybenzone might cause harm. Unfortunately, such “population-wide” evidence will be incredibly challenging to collect. Epidemiology studies that attempt to examine such relationships often fail due to the ecological fallacy (i.e., drawing conclusions about individuals based on evidence collected for a group, in this case, concluding that oxybenzone is safe for individuals based on a failure to obtain a “population-wide signal” of harm). Furthermore, it is almost impossible to find a population of individuals without exposure to oxybenzone to serve as an “unexposed control group”; in 2011, the chemical was detected in the urine of 98% of non-pregnant women and 100% of pregnant women [80]. Even individuals that report “never” using sunscreen have detectable levels in their urine, indicating that exposures come from a wide variety of sources [81]. Of course, if such a population-wide signal of harm were identified, that would represent a massive public health failure; the possibility of such catastrophic outcomes creates an even stronger case for precaution [82].

In spite of these limitations, an increasing number of human studies suggest associations between oxybenzone and harm. These studies examine exposures and health outcomes at the level of individuals, finding “signals” of harm that have unfortunately been dismissed by some in the dermatology community because the studies do not examine the population in entirety. These findings should not be ignored in a quest for a “perfect” human cohort. With the evidence that is currently available, the principle of “first do no harm” disqualifies oxybenzone due to its maleficence (Fig. 3).

### Principle of justice

*Hawaii is definitely on the cutting edge by banning these dangerous chemicals in sunscreens.*

Hawaii State Senator Mike Gabbard

The *Principle of Justice* in environmental health demands that individuals and communities share “justly and equally both burdens and benefits” and that “societies follow fair procedures” in making and implementing policy decisions concerning the environment [38]. In the Rawlsian approach of justice – where justice is consistent with fairness [83] – and in the extension of Rawls’s principles to health care, unequal health burdens are seen as unjust when social determinants of health such as education or job opportunities are also unjustly distributed [84].

There are many benefits of coral reefs to humans, including tourist spending, food sources, coastal protection and populations that live near these locations can enjoy them [85]. Yet those unique ecosystems are subjected to unprecedented worldwide losses. Oxybenzone is one of several contributors to coral bleaching, brought to reefs indirectly from residential and municipal wastewaters or washed off the skin of swimmers and beachgoers. Beach tourism has become ever more popular; for example, the annual number of visitors to the State of Hawaii – whose islands are surrounded by coral reefs - increased from 6.7 million to 10.4 million between 1999 and 2019 [86, 87]. Coastal pollution with oxybenzone results in an unequal distribution of an environmental burden. After Hawaiian residents realized the distributive injustice of this burden, they exercised their right to procedural justice. In 2018 the State Senate voted to ban sunscreen products containing oxybenzone and octinoxate from being distributed or sold in Hawaii, taking effect in January 2021 [88].

The Hawaii case study provides an example of a specific population acting meaningfully against environmental pollution. Yet, a situation where governing legislators rule confidently and effectively against a polluting agent is an exception rather than a norm. Typical stories of disproportionate pollution in communities commonly end – if resolved at all – in cumbersome repressive legal actions and public budgets paying for the consequences of the pollution [89].

Ideally, distributional injustice is prevented. If it cannot be prevented, the disadvantaged community should have the means to decide on the burden experienced through procedural justice or to be compensated for the unequal and unjust distribution. Concerted efforts are needed to reduce the unjust, distributional burden of pollution on the global scale (Fig. 3).

Oxybenzone provides one additional angle to the *Principle of Justice*: this chemical has been detected in the bodies of individuals across the globe, regardless of their personal decisions or reported use of chemical sunscreens [81]. Above all, neonates are born having already been exposed to chemical sunscreens [80]. Polluting our descendants raises the question of intergenerational justice, a topic described above.

### Principle of community

*“The innocent greed of the affluent may easily be the most threatening ecological time bomb.”*

*E. Kohak* [46]

The Principle of Autonomy – defined as “acting on the basis of choices guided by values and principles that one accepts as one’s own” [90] - is the paramount guiding principle in medical practice and bioethical theory. But public health practice typically applies a number of restrictions on this fundamental principle because in challenges posed by public health, the “interests of the community” override individual choices if personal preferences pose a risk to health or safety of the community [91]. Examples of what we have dubbed the *Principle of Community*, where the health of the community is seen as a vital priority, include restrictions on smoking in public or shared spaces due to the risks of secondhand smoke [92], required vaccination for the sake of herd immunity [93], or mask mandates in the case of a pandemic caused by a respiratory virus. Environmental health practice builds on similar premises of community kinship, where the focus is on the interests of environment and human health even when the benefits to the community conflict with individual preferences. The concept of the ‘tragedy of the commons’ relates to this point, because the benefits received by the individual (or a specific industry) can detract from the health of the whole [94].

To illustrate the environmental health conflict, Kohak (p.9 [46]) uses the example of a retired clergyman whose hobby is to fly surplus fighter jets, arguing that it is his *right* to engage in the activity, even while he acknowledges the detrimental environmental impact of fighter jet engines. Kohak frames such reasoning as an “innocent greed of the affluent” and asserts that similar forms of argumentation are not uncommon in human-environment conflicts [46]. The “innocent greed” argument represents one of many hypothetical explanations for individual choices to use sunscreens containing oxybenzone despite their adverse effects on the environment. Users may think, “It is my right to use this sunscreen, even if it contributes to environmental harm.” For those reasons, human-environment conflicts are often addressed with regulatory action to restrict autonomous decisions.

Although the “innocent greed” argument is certainly important to consider, lack of awareness reflects another, more likely, concern because the general public is not typically expected to judge the risks associated with the use of synthetic chemicals or exposure to environmental pollutants. Instead, experienced professionals – scientists, public health professionals, and regulators – are tasked with using expert judgement to tackle the complex problems of environmental and human risk assessment. In other words, regulatory agencies and their employees are tasked to *prevent* the necessity of the consumers’ dilemma.

Unfortunately, autonomous choices cannot always be respected when environmental health is at stake or when the impacts of autonomous choice are potentially disastrous [82]. Such choices should not even be expected when addressing issues that are as complex and complicated as the case of chemical safety evaluations. While legislative regulations are not without risk (e.g., what has been described as the “slippery slope of regulations [95]), the case of oxybenzone shows that that under the *Principle of Community*, some groups may need to override autonomous (individual) decisions to ward off the magnitude of environmental, societal and/or health costs that are attributed to the collective impact of individual acts (Fig. 3).

### The principle of precautionary substitution

*The term ‘precautionary principle’ can be traced to the German word* Vorsorgeprinzip*. An alternative translation of this word might be the foresight or ‘forecaring’ principle—emphasizing anticipatory, forward-looking action rather than reactive impeding of progress.*

*Tickner, Kriebel & Wright* [96]

The *Principle of Precautionary Substitution* cautions *against* replacement of harmful chemicals if such a replacement introduces another equally or more harmful chemical. In recent decades, a number of everyday or industrial chemicals were disfavored and substituted with different chemicals that were later found to be harmful. For example, lead arsenate, an insecticide comprised of two heavy metals, was used on deciduous trees (including many apple orchards) [97] until it was replaced by the infamous DDT. The impact of Rachel Carson’s *Silent Spring* brought the use of DDT in U.S. agriculture to an end and organophosphate pesticides quickly replaced organochlorines. Yet, with time, many of these were also found to be toxic and were later banned from residential use (although malathion is still heavily used for mosquito control and in agriculture) [98, 99]. Then came the neonicotinoids, insecticides now described as “bee neurotoxins”; three of them are now “severely restricted” in the EU and only three are permitted in Canada [100, 101]. The newest pest control inventions target genetic information, e.g. RNAi (RNA intervention) pesticides, but whether the “blissful enthusiasm that accompanies every new advance in modern technology and medicine” [102] will lead to a similar disillusionment with these technologies remains to be seen.

Precaution pertaining to oxybenzone requires us to examine two opposing sides: the concern about *not using* chemical sunscreens which is perceived as increasing the risk of skin cancer [103], and concerns about *using* chemical sunscreens, which increases environmental pollution and adverse health effects in human and non-human species. Both these facets are rarely examined together and often fail to address the relevant issues, e.g., a frank accounting of the effectiveness of sunscreens in skin cancer prevention, as well as the full magnitude of understanding the many ways chemical sunscreens impact life on the planet. The former issue was addressed above in the *Principle of Beneficence* and we turn to the latter in this section.

Extensive evidence collected in wildlife and from experimental studies suggests that oxybenzone harms a wide range of species including bacteria, algae, plants, fish and mammals (Fig. 2). When the impact of oxybenzone on coral reefs galvanized concerns and induced a negative public response, attention shifted towards UV protecting alternatives. Novel technological-chemical solutions (e.g., variants of chemical sunscreens including mineral-based sunblock improved with nanotechnologies), and emerging chemical solutions (e.g., benzotriazoles, a newer family of UV stabilizing chemicals added to consumer products) represent a marketing opportunity, but they also raise red flags around human health and sustainability. For example, titanium dioxide nanoparticles penetrate human skin in certain formulations and emerging evidence points to their potential toxicity [104, 105]. Similarly, UV-328, a benzotriazole UV stabilizer added to many plastics is proposed to be added to the Stockholm Convention’s list of persistent organic pollutants [106] and other benzotriazoles such as HDBB and UV-324 induce toxicity in aquatic species and act as endocrine disruptors in both fish and mammals [107].

To address the issue of regrettable replacements, several distinct actions have been proposed. First, it is possible to regulate chemicals by *groups or classes*. An international effort on “Substances depleting the Ozone Layer” – The Montreal Protocol – provides an example of successful regulation of a chemical class based on the type of harm inflicted by chemicals [108]. Alternatively, chemicals could be classified and regulated based on their intended purpose. The concept of “essential” vs. “non-essential” use suggested for classification of per- and polyfluoroalkyl substances (PFAS) would enable regulators to identify and phase-out the most non-essential uses [109]. Second, the price of a product producing unintended harms could more fully reflect those detrimental costs known as externalities. Economists specialized in sustainability (i.e., the discipline of Ecological Economics) can apply various tools for internalizing costs associated with disposal and pollution and can quantify the benefits obtained by industries utilizing natural ecosystems [110]. Accountability of the markets for using and overusing natural resources differs from recent economic theories (and practice) but a society in which individuals and public budgets cover health and societal costs associated with profit-driven polluting producers is not sustainable on the scale we have achieved today.

## Conclusions

Environmental pollution with thousands of inadequately tested synthetic chemicals is among the many urgent threats that challenge human health and environmental sustainability. While the Paris Agreement takes action against greenhouse gas emissions, a similar international vision on anthropogenic pollution has yet to be formed.

We have offered six principles that are relevant for environmental health decision-making (Fig. 1); these principles illustrate the complexity of the problem of environmental chemical pollution and should be considered when searching for solutions. A reliance on principles provides guidance to evaluate compounds that present a patchwork of risks and benefits in both societal and scientific contexts. Thus, the case of oxybenzone lends an excellent opportunity to apply these six principles of environmental health to a specific chemical that is used in consumer products to meet a specific need (protection from UV-induced damage), but with costs to human and environmental health that have been largely unexplored (Fig. 3). With the framework of principlism, it becomes clear that even for a chemical with relatively moderate stakes (compared to the wide spectrum of highly persistent and highly accumulative compounds that have been released to the environment), action is required to address the limits of the biosphere’s regenerative rates.

## Data Availability

Not applicable.
